# Malariometric indices from Iganga, Uganda: baseline characterization in preparation of GMZ2 vaccine trial

**DOI:** 10.1186/1756-0500-7-793

**Published:** 2014-11-08

**Authors:** Mark Kaddumukasa, William Buwembo, Musa Sekikubo, Halima Naiwumbwe, Fatuma Namusoke, Stephen Kiwuwa, Brenda Oketch, Ramadhani Noor, Roma Chilengi, Edison Mworozi, Fred Kironde

**Affiliations:** Makerere University College of Health Sciences, Kampala, Uganda; Makerere University College of Natural Sciences, Kampala, Uganda; Harvard School of Public Health, Boston, USA; Centre for Infectious Research in Zambia (CIDRZ), Lusaka, Zambia; St. Augustine International University (SAIU), Kampala, Uganda

**Keywords:** Malaria episodes, Parasitaemia, Surveillance, Iganga, Uganda

## Abstract

**Background:**

Malaria still remains the leading cause of childhood morbidity and mortality in Uganda. Interventions like malaria vaccines which reduce the malaria burden are needed in malaria endemic communities. There is need to establish baseline characteristics in vaccine trial study sites. This study determined the following baseline malariometric indices: spleen rates, bed net use, malaria parasitaemia and malaria episodes in an inception cohort of children aged 12 – 60 months in Iganga district, Uganda.

**Methods:**

In a longitudinal cohort study, 748 children were enrolled with 397 in an active follow up arm and 351 in a passive arm. The children in the two arms were followed for 6 months to determine the incidence of malaria episodes.

**Results:**

The overall baseline spleen rate was 8.2% (61/748) among the study participants. Of the households surveyed, about 36% reported using bed nets and almost 30% of the users had insecticide-treated nets. 274 (36.6%) of the study participants had a history of fever in the past 24 hrs at the time of the baseline survey. All participants had a peripheral blood smear for malaria parasites done at enrollment with 76.8% having the asexual form of malaria parasites. The malaria episodes per child per year were 1.5 and 0.79 in the active and passive follow up arms respectively.

**Conclusions:**

There is a high prevalence of malaria asexual parasitaemia in children below five years. The bed net usage still remains low among this population. These baseline malariometric indices have important implication for malaria control interventions.

## Background

Malaria still remains one of the leading global health problems with more than half a million death per year in Africa, occurring in children below 5 years of age [1]. Malaria control in resource limited settings has been hindered mainly by increasing resistance to commonly used anti-malarial drugs and under utilization of preventive measures [2]. The remedy for Africa remains in drug development (artemisinin-based combination treatments), preventive interventions (long-lasting, insecticide-treated bed nets), improved diagnosis (rapid diagnostic tests), community mobilization and development of effective vaccines [3–5]. However, understanding of the epidemiology of malaria in malaria clinical trial sites is needed to evaluate the impact of control interventions. The burden of malaria can be studied using longitudinal studies which employ the extended active follow-up methods to guide a comprehensive evaluation of malaria infection outcomes and incidence [2]. Well-defined cohorts from the sub – Saharan Africa have been instrumental in characterizing the epidemiology of malaria, exploring the acquisition of immunity, and demonstrating the effectiveness of control interventions such as insecticide-treated bed nets, intermittent preventative treatments and vaccines [6–11].

In 2009, the African Malaria Network Trust (AMANET) received a grant from the European and Developing Countries Clinical Trials Partnership (EDCTP), to strengthen capacity in selected African Research institutions in conducting malaria vaccine trials. Makerere University, College of Health Sciences, Uganda, was one of the beneficiaries of the sub-grants awarded by AMANET, but baseline malariometric indices for site characterization to aid in recruitment and follow up of the volunteers were not available. The objective of the current study was to describe the baseline malariometric indices including spleen rate, bed net use, in Iganga district, Uganda. Results from this study were used together with other data [12] to provide guidance in conducting as well as setting up a cohort for GMZ2 [13] vaccine trial.

## Methods

### Description of study site

Our study area in Iganga district comprised of six villages located in the neighborhood of Iganga district hospital. Iganga district is predominantly rural with one government hospital, 10 smaller governmental health facilities, three non-government facilities, 122 drug shops and clinics. Iganga hospital is located about 120 kilometers east of Kampala, the capital city of Uganda. The total population of Mayuge/Iganga is about 70,000 with the under five population being 11,000. Like elsewhere in Uganda, malaria is one of the leading health problems. The climate in Iganga is equatorial, with *Plasmodium falciparum (P. falciparum)* as the predominant malaria species. Malaria transmission occurs throughout the year but peaks are seen following maximal vector breeding after the rains: periods April to June and September to December. The annual entomologic inoculation rate (EIR) is not known, but is reported to be >500 infective bites/person/year in the neighboring district of Tororo [14].

The study site is a typical rural area, primarily residential, characterized by a high population density with low-income single or two-roomed housing units. The local economy is predominantly dependent on petty commercial activities and small-scale farming. The principal mosquito vector is *Anopheles gambiae sensu strictu* and their analysis for resistance markers is still ongoing.

### Recruitment and demographic survey

A representative cohort of 748 children aged 1–5 years participated in a six month longitudinal study to determine the malaria episodes, the normal reference hematological and biochemical ranges which have been reported elsewhere [12] and also provide recruitment for a malaria vaccine study. The sample size of 300 per arm was estimated based on assumptions of malaria incidence of 15% in the active arm, with 95% confidence interval, and ability to detect a vaccine efficacy of 30%. However, assuming a 15% loss to follow up and non response for recruitment into the vaccine study, 350 children were estimated for each arm. The participant recruitment provided a cohort of children for subsequent enrolment into a vaccine trial. Prior to the start of the study, investigators met with administrative government representatives to inform them of the study, its objectives and explain the methodology. Priority villages were selected and earmarked in consultation with the district officials as these were noted to have a large burden of malaria related problems. Through local village meetings, residents from each village were informed about the intended project objectives. Community consent was sought before household screening for potential participants and subsequently individual consent was obtained from each participant. Using a systematic random sampling method, the initial household located in the centre of each village was selected as a starting point and every third household was approached for potential enrolment and screening based on the eligibility criteria. If the sampled household did not have an eligible participant then the next household was approached. A resident of the village was defined as a person anticipated to be staying primarily within that location for the next 6 months.

Over a two month period, teams of four study personnel systematically covered the entire area of the six villages on foot to identify and enumerate all households with help of the local council official of that village. A household was defined as any single permanent or semi-permanent structure acting as the primary residence for a person or group of people. The enrolled households were assigned sequential unique numbers, written on a visible label fixed above the doorway, and residents were given a household identification card. After enumeration, study personnel asked the household head or guardian from each household for verbal consent to participate in a brief demographic survey. Using a standardized questionnaire, demographics and malaria indicator information was collected. If no adult resident was available, that household was excluded from the survey.

### Recruitment of the cohort

Household level recruitment was for one eligible child from each household. If a household had more than one eligible child then a raffle was conducted to enroll only one child. From January 2010 to February 2010, experienced home visitors approached households to systematically recruit from the previously generated list. Using handheld GPS receivers they recorded households’ coordinates and a brief description of the study was provided in the appropriate language to parents and/or guardians.

### Screening and enrollment

Interested potential participants were screened sequentially until the desired sample size was achieved. Initial assessment of eligibility was conducted using a selection criteria of; 1) age one to five years; 2) agreement to come to study clinic for any febrile episode or illness; 3) agreement to avoid medications administered outside the study; 4) agreement to remain in study area during the six months follow up; 5) absence of known chronic disease and 6) written informed consent provided by parent or guardian. Children who passed initial screening were assigned study numbers and underwent a history and physical examination including temperature, height weight measurement and abdominal examination. Severely malnourished children [15], were excluded. Hemoglobin concentration was measured and those with hemoglobin levels less than 9 g/dl were referred to the district hospital for further management. Presence of bed net was verified by direct observation at home. All children had a screening RDT performed and a thick blood smear performed which was read later. Children having a history of fever in the past 24 hours, a tympanic temperature of 38°C or higher and a positive thick blood smear at the time of initial screening were treated with Artemether – Lumefantrine (according to their weight for 3 days) according to the Uganda national treatment guidelines. A clinical examination was performed to assess the presence and size of the spleen. Follow-up started when children fulfilled all of our selection criteria and were free of symptomatic malaria. The villages were divided by convenience into active (nearby villages) and passive (further) villages.

### Passive case detection

Children in the passive arm were visited every month by trained field workers to document absences from the study site and find out how they had been in the past four weeks. Parents/guardians were encouraged to bring their child to the malaria clinic if the child was unwell.

### Active case detection

Children in the active follow up arm were visited twice a week by field workers. A standardized questionnaire was administered for collecting information regarding illnesses that had occurred since the last visit, use of health care facilities and medications used. At each visit, the tympanic temperature was recorded using a digital thermometer. When fever (tympanic temperature of ≥38°C) or history of recent fever (within the last 24 hours) was observed or reported for any study child, a rapid diagnostic test (RDT, OPTIMAL®) and microscopy of a stained blood smear were used to screen for malaria and to confirm the presence of malaria parasites respectively. Children found with any peripheral asexual malaria parasitaemia in their blood were treated at the study clinic accordingly following the Uganda national treatment guidelines.

### Microscopy

Thick blood smears were stained with 2% Giemsa for 30 minutes. Parasite density was estimated by counting the number of asexual parasites per 500 white-blood cells and calculating parasites per μL, using the actual white blood cell count of the subject. A smear was judged to be negative if no parasites were seen after examination of 100 high-powered fields. A second microscopist, who was unaware of the results of the first reading, re-read all slides. A third reviewer resolved discordant results. The presence of sexual forms of the parasites was also determined during slide readings.

### Data management and analysis

Baseline data were collected in the field on written forms, verified, checked by investigators and entered into the *Open Clinica* ( https://www.openclinica.com) database. Double entry verification from paper questionnaires was performed to assure accuracy. Categorical variables were compared using the chi-square test and continuous variables using a two-sample t-test. A p-value <0.05 (two-tailed) was considered statistically significant. Analysis was done using SPSS version 10.0 (SPSS, Chicago, IL, USA).

### Ethical issues

Written informed consent was obtained from the parent/guardian(s) of children for their participation in the cohort study. The study was approved by Makerere University School of Medicine, Research and Ethics Committee 2009/172 and by the Ugandan National Council of Science and Technology (records no. HS 765).

## Results

A total of 1161 households were screened, of which 748 were enrolled, 374 (32.2%) households had no children aged less than 5 years, while 39 (3.4%) declined to consent for the study.

Table 1: shows the demographics characteristics of study cohort (n = 748). The mean age was 2.3 ± 1.2 years. Majority of the respondents were from the Basoga tribe which is the predominant tribe in Iganga district. The overall spleen rate at baseline was 8.2% (61/748) among the study participants. There was no significant difference between participants in the active and passive arms in age, sex and temperature. However the spleen rate was significantly different between these two arms (P = 0.002).

**Table 1:** **Demographic characteristics of study participants**

Characteristics	Arm					
	Active		Passive		Chi-square	p-value
	(N = 397)	%	(N = 351)	%		
**Age in years**						
Less than 1	127	32.0	107	30.5		
2-3	193	48.6	180	51.3		
4-5	77	19.4	64	18.2		
**Sex**						
Male	221	55.6	191	54.4		
Female	176	44.3.4	160	45.6		
**Ethnicity**						
Basoga	392	98.7	341	97.2		
Others	5	1.3	10	2.8		
**Slept in bed net**						
Yes	143	36.0	130	37.1	0.08	0.777
No	254	64.0	221	62.9		
**Was the bed net treated**						
Yes	109	76.8	117	90.0	9.07	0.002*
No	34	23.8	13	10.0		
**Fever in the past 24 hours**						
Yes	153	43.6	121	34.5	1.330	0.248
No	244	56.4	230	65.5		
**Spleen rate**						
Yes	21	5.3	40	11.4	9.211	0.002*
No	376	94.7	311	88.6		
**RDT results**						
Positive	265	66.8	192	54.7	10.483	0.001*
Negative	132	33.2	159	45.3		
**Malaria asexual parasitemia**						
Positive	294	70.1	281	80.1	5.057	0.025
Negative	103	29.9	70	19.9		

^*^Statistically Significant.

### Use of bed nets

Among the study population, the overall bed net usage among the two arms was reported at 36.4% (273/748) of whom 82.8% (226/273) reported that they were using insecticide treated nets. Overall, 64% (475/748) of the study participants reported that they did not own bed nets or had not used one during the preceding night before the baseline survey. However, there was no significant difference in bed net usage or ownership between the active and passive follow up groups (P = 0.777).

### Malaria

**Figure 1:**
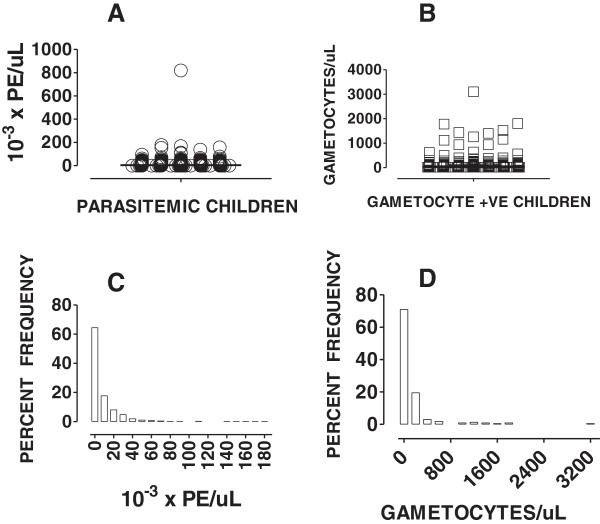
**Endemicity of malaria at the malaria vaccine site of Iganga.** To determine the endemicity of malaria at the malaria vaccine site of Iganga, 748 children aged 1 to 5 years were screened for history or presence of fever and confirmed by microscopy of stained blood smear. Majority (76.8%) of the children had *P. falciparum* in the blood. Except one child, all children had <180,000 PE/uL (see **A, C**). The median gametocytemia was 40 /uL (IQR: 20–100; see **B & D**).

Among the study participants 274 (36.6%) reported history of fever in the past 24 hours before the baseline survey was conducted. Overall 61% (457/748) of the study subjects had a positive screening RDT test at enrolment. Fifty eight percent (265/457) of those with a positive RDT were from the active arm. However, out of the 274 subjects with history of fever in the preceding 24 hours, only 66.4% (182/274) had a positive screening RDT malaria test at enrolment. And, the thick blood smears showed that 76.8% (575/748) of the study participants had asexual *P. falciparum* malaria parasites in their peripheral blood smears at baseline screening during recruitment (Table 1). Nearly, 99% (569/575) of these subjects had a mono-infection of *P. falciparum* while 1% (6/575) had a dual infection with both *P. falciparum* and *P. vivax*. While the median asexual parasite density (Figure 1A and C) was 4224 Parasitized erythrocytes (PE)/uL (IQR : 1,005-14,040) and the children with asexual parasite density greater than 5000 PE/uL comprised 35.5%. The gametocyte carriage rate was 30.4% with median gametocytemia of 40 gametocytes/uL (IQR: 20 –100; Figure 1B). Over 90% of children with gametocytes, carried less than 400 gametocytes per uL of blood (Figure 1D).

### Malaria incidence and episodes

**Figure 2:**
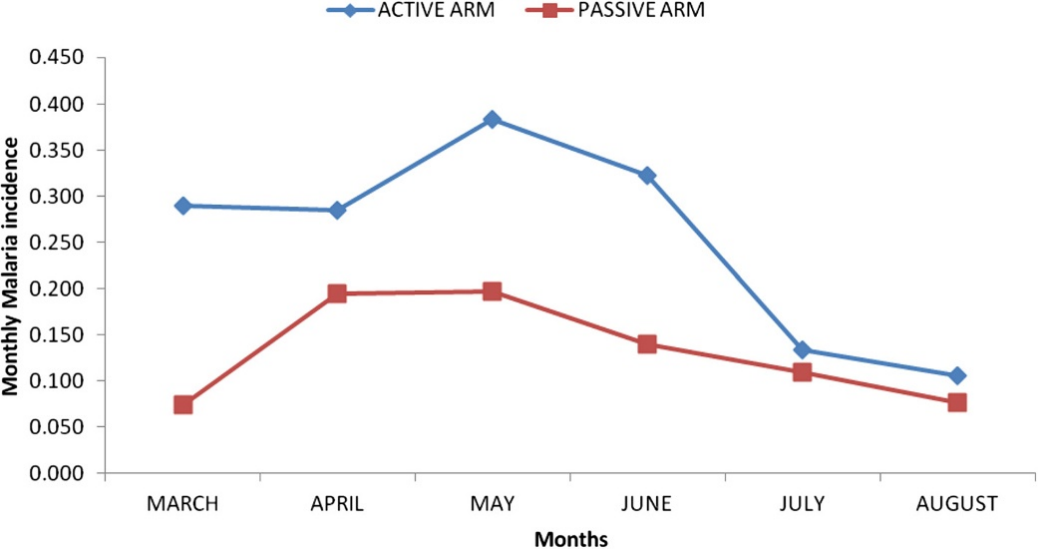
**Monthly malaria incidences for the six month follow up period.** The malaria incidences were plotted against months for the six months the study was carried out. The active arm had a higher incidence throughout the six months and the highest peaks were in May and April in the active and passive arms respectively.

**Figure 3:**
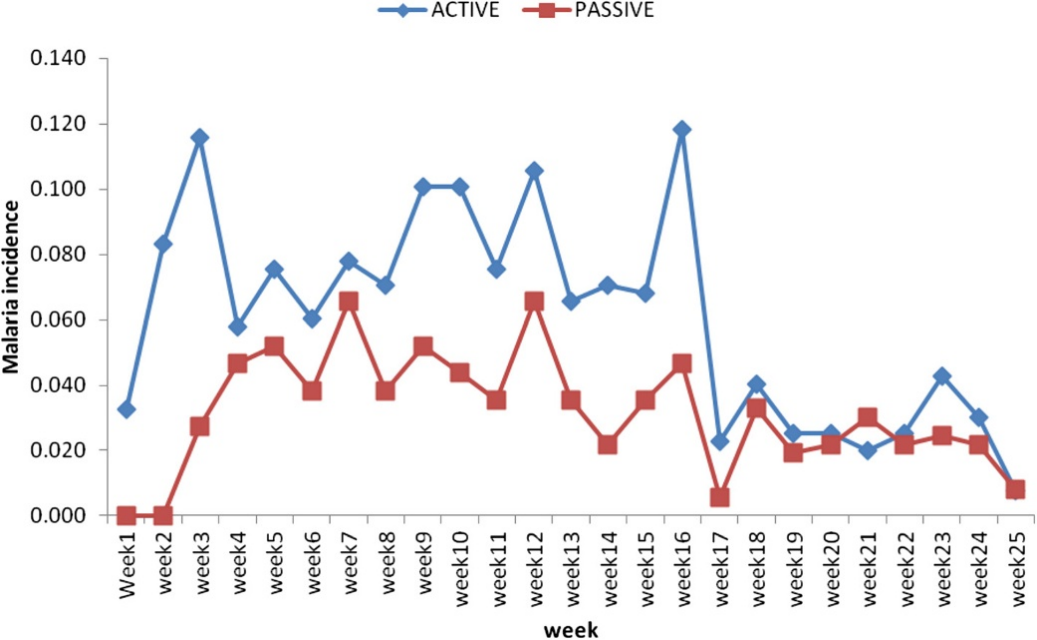
**Weekly malaria incidences for the 6 months follow-up period.** The incidences were plotted against weeks for the months the study was carried out. The active arm had a higher incidence on a weekly basis for most of the months with an overlap during the last one and half months.

Total malaria cases for the 6 months follow-up period (March – August 2010) were 892 among the study participants (Table 2). Of these 603 (67.6%) were among the active arm, while 289 (32.4%) occurred in the passive arm group. The peaks for the most malaria cases seen at the malaria clinic were in the months of May for the active arm and April for the passive arm. The weekly clinic attendances were higher for the active arm as compared to the passive arm during the follow up period except during week 2 (Figures 2 and 3). The malaria episodes that occurred during the follow up period were 1.5 and 0.79 per child per year for active and passive arms respectively. Children aged 2–3 years in both arms had a higher malaria incidence compared to the other age groups (Table 3). Of the 748 study participants, 311 (40.9%) had no malaria episode during the study period, 200 (26.3%) had an episode, 124 (16.3%) had two episodes, 71(9.3%) had three episodes, 28 (3.7%) had four episodes, 14 (1.8%) had five episodes, 7 (0.9%) had six episodes, 2 (0.3%) had eight episodes. One child (0.1%) had seven episodes of malaria; another had nine episodes while a third had 16 episodes during the follow up period.

**Table 2:** **Total malaria cases for the 6 months follow-up period (March – August 2010) according to the study arm**

Month	Active (n = 397)		Passive (n = 351)		Total
	No	%	No	%	
March	115	81.0	27	19.0	142
April	113	61.4	71	38.6	184
May	152	67.9	72	32.1	224
June	128	71.5	51	28.5	179
July	53	57.0	40	43.0	93
August	42	60.0	28	40.0	70
**Total**	**603**	**67.6**	**289**	**32.4**	**892**

**Table 3:** **Malaria incidence according to the age**

Age group	Active (n = 397)		Passive (n = 351)	
	No. of cases	Incidence	No. of cases	Incidence
0 – 1	62	0.16	54	0.15
2 – 3	413	1.04	179	0.49
4 – 5	128	0.32	56	0.15
**Total**	**603**	**1.52**	**289**	**0.79**

## Discussion

Longitudinal studies in well-defined cohorts are critical to improving our understanding of the malaria incidence and associated factors before interventions can be introduced. In the current study, a representative cohort of children from a rural setting where malaria is highly endemic was enrolled.

The overall prevalence of malaria parasitaemia at baseline in the study population was 76.8%, this suggests that Iganga is a holoendemic area. This corroborates the general observation that the under fives are more vulnerable to the disease in areas of high transmission [16]. However it is higher than the reported malaria prevalence of 56% in Eastern Uganda [17] probably due to the difference in timing of the surveys. Our survey was conducted during one of the high transmission periods of March to August; this might also explain the high levels. Naturally acquired immunity builds up in older children following repeated exposure to the parasite and is manifested by lower parasite densities and fewer clinical malarial episodes than in younger children and less exposed under fives [18]. In our study children aged 2–3 years had a higher incidence in both groups. This may be due to the increased exposure due to the waning maternal antimalarial antibodies, this corroborates earlier reports which showed children aged 2 – 4 years to have an increased risk of cerebral malaria [19].

However, the prevalence of those with symptoms of fever was 36.6% at the baseline survey. This further highlights the fact that some children may have asymptomatic malaria. *P. falciparum* was the predominant species of malaria parasite identified in this study; with majority 99% of the children having a *Plasmodium falciparum* mono-infection. The high prevalence of malaria and *P. falciparum* mono infection noted in this study is an indication of a worsening malaria situation around the study area which might be indicative of poor access or adherence to effective control measures such as the use of insecticide treated nets (ITNs). This is similar to earlier findings reported from other study sites within the region [20]. Only 36.4% of the study children reported using bed-nets of whom only 30% were using insecticide-treated nets. This is lower than the reported 33% of ITN usage in Eastern Uganda [17]. This study population is predominantly poor and rural and this might affect the probability of owning a bed net as well.

The overall spleen rate in this study is 8.2%. This supports the fact that children aged less than five years have a more pronounced immunological response (including splenic enlargement) to malaria [21]. However, other hematological diseases and factors responsible for spleen rate were not assessed in this study. The higher spleen rate in the passive arm was probably due to the persistent parasitemias as the children were only brought to the clinic for treatment when they fell ill. Persistent parasitaemia from treatment failure is known to contribute to splenic enlargement, in earlier reports [22]. A study in Nigeria has shown that the spleen rate may reduce as prompt access to effective treatment to malaria improves [23].

The episodes of malaria in the active follow up arm was twice that of the passive arm during the period of observation. This corroborates other reports which have used similar methodology for malariometric indices [24]. This might be due to weekly reminders of sending children to the clinic whenever they felt unwell as compared to those in the passive arm. Some of the malaria infections in the passive arm may have been treated and not reported to the clinic. The proportion of children with malaria reduced steadily with increasing episodes of the infection. The susceptibility of some children to more malaria episodes as the one who had up to sixteen episodes compared to others is unexplained. This may be due to differences in the host genetics which were not part of this study but will need further studies to elucidate.

## Conclusions

This study shows that malaria remains a major health problem among children aged less than five residing in Iganga district, eastern Uganda. The bed net usage still remains low among this population. This baseline study provides the important malariometric data required for vaccine intervention studies to determine their efficacy in our setting.
